# Supplementary material for: Interventions Provided by Physiotherapists to Prevent Complications After Major Gastrointestinal Cancer Surgery: A Systematic Review and Meta-Analysis
Source: Cancers (Basel). 2025 Feb 17;17(4):676. doi: 10.3390/cancers17040676 (PMC11853706; doi:10.3390/cancers17040676)
Supplement: Supplementary file 1 [file cancers-17-00676-s001.zip › SR supplementary excluded studies V2 SW.pdf]

Supplementary material 2: Table 3S Excluded studies

256 papers failed to meet the inclusion criteria at the full-text screening stage. These papers and the reason for exclusion are presented in the table below. These papers are presented according to the hierarchical methods in which the exclusion reasons were applied from 1) Wrong study design 2a) Wrong patient population (eg. All cholecystectomy or bariatric surgery) 2b) Unable to ascertain if > 50% of population having surgery due to gastrointestinal cancer 3) Wrong intervention 4) Wrong comparator (eg. One respiratory intervention being compared to another active respiratory intervention 5) Wrong outcome measures 6) Intervention delivered by health professionals other than physiotherapist 7) Study not in English

Study	Exclusion reason
Abdelfatah, E.; Ramos-Santillan, V.; Cherkassky, L.; Cianchetti, K.; Mann, G. High Risk, High Reward: Frailty in Colorectal Cancer Surgery is Associated with Worse Postoperative Outcomes but Equivalent Long-Term Oncologic Outcomes. *Annals of Surgical Oncology* **2023**, *30*, 2035-2045, doi:10.1245/s10434-022-12970-7.	Wrong study design
Agrelli, T.F.; Ramos, M.D.; Guglielminetti, R.; Silva, A.A.; Crema, E. Preoperative Ambulatory Inspiratory Muscle Training in Patients Undergoing Esophagectomy. A Pilot Study. *International Surgery* **2012**, *97*, 198-202, doi:10.9738/cc136.1.	Wrong study design
Akkaya, S.B.; Karacabay, K. Investigation of the Effect of Ambulation Time on Patient Outcomes Anxiety and Depression. *Journal of Basic and Clinical Health Sciences* **2022**, *6*, 217-224, doi:10.30621/jbachs.1018224.	Wrong study design
Alfonsi, P.; Schaack, E. Accelerated Postoperative Recovery after Colorectal Surgery. *Journal De Chirurgie* **2007**, *144*, 191-196, doi:10.1016/s0021-7697(07)89513-5.	Wrong study design
Bates, A.; West, M.A.; Jack, S.; Grocott, M.P.W. Preparing for and Not Waiting for Surgery. *Current Oncology* **2024**, *31*, 629-648, doi:10.3390/curroncol31020046.	Wrong study design
Booth, A.T.; Curran, T. Preoperative Optimization and Enhanced Recovery for Colorectal Surgery. *Seminars in Colon and Rectal Surgery* **2021**, *32*, doi:10.1016/j.scrs.2021.100827.	Wrong study design
Bruns, E.R.J.; Argillander, T.E.; Schuijt, H.J.; van Duijvendijk, P.; van der Zaag, E.S.; Wassenaar, E.B.; Gerhards, M.F.; Consten, E.C.; Buskens, C.J.; van Munster, B.C.; et al. Fit4surgerytv at-Home Prehabilitation for Frail Older Patients Planned for Colorectal Cancer Surgery a Pilot Study. *American Journal of Physical Medicine & Rehabilitation* **2019**, *98*, 399-406, doi:10.1097/phm.0000000000001108.	Wrong study design
Burke, M.; Dolan, A.; Church, G.; Clark, M.; Edwards, J.; Moug, S. Targeted post-op physiotherapy improves mobilisation and reduces respiratory complications in Emergency Laparotomy patients. *British Journal of Surgery* **2020**, *107*, 101-101.	Wrong study design
Canet, J.; Mazo, V. Postoperative pulmonary complications. *Minerva Anestesiologica* **2010**, *76*, 138-143.	Wrong study design
Cavallaro, P.M.; Milch, H.; Savitt, L.; Hodin, R.A.; Rattner, D.W.; Berger, D.L.; Kunitake, H.; Bordeianou, L.G. Addition of a Scripted Pre-Operative Patient Education Module to an Existing Eras Pathway Further Reduces Length of Stay. *American Journal of Surgery* **2018**, *216*, 652-657, doi:10.1016/j.amjsurg.2018.07.016.	Wrong study design
Celli, B.R. Perioperative Respiratory Care of the Patient Undergoing Upper Abdominal-Surgery. *Clinics in Chest Medicine* **1993**, *14*, 253-261.	Wrong study design
Chow, J.J.L.; Teo, Z.H.T.; Acharyya, S.; Natesan, S.; Cheong, S.K.; Tony, S.; Ong, Y.W.; Li, Y.J.; Wang, B.; Chai, J.Y.; et al. Recovery of surgery in the elderly (ROSE) program: The efficacy of a multi-modal prehabilitation program implemented in frail and pre-frail elderly undergoing major abdominal surgery. *World Journal of Surgery* **2024**, *48*, 48-58, doi:10.1002/wjs.12016.	Wrong study design

Colucci, D.B.B.; Fiore, J.F.; Risso, T.T.; Paisani, D.M.; Stuchi, T.P.; Shiguemichi, L.H.; Chiavegato, L.D.; Faresin, S.M. Is the Impairment of Cough Efficacy Associated to the Development of Postoperative Pulmonary Complications after Upper Abdominal Surgery? *American Journal of Respiratory and Critical Care Medicine* **2009**, *179*.	Wrong study design
Daksla, N.; Nguyen, V.; Jin, Z.S.; Bergese, S.D. Brain Prehabilitation for Oncologic Surgery. *Current Oncology Reports* **2022**, *24*, 1513-1520, doi:10.1007/s11912-022-01312-1.	Wrong study design
Davies, J.M. Preoperative Respiratory Evaluation and Management of Patients for Upper Abdominal Surgery. *Yale Journal of Biology and Medicine* **1991**, *64*, 329-349.	Wrong study design
Davis, S.P. Incentive Spirometry after Abdominal Surgery. *Nursing Times* **2012**, *108*, 22-23.	Wrong study design
Delay, J.M.; Jaber, S. Respiratory Preparation before Surgery in Patients with Chronic Respiratory Failure. *Presse Medicale* **2012**, *41*, 225-233, doi:10.1016/j.lpm.2011.08.007.	Wrong study design
do Nascimento, P.; Koga, F.A.; Modolo, N.S.P.; Andrade, S.; Braz, L.G.; El Dib, R. Incentive Spirometry for Prevention of Postoperative Pulmonary Complications in Upper Abdominal Surgery. *European Journal of Anaesthesiology* **2014**, *31*, 79-79.	Wrong study design
Dodia, B.K.; Shetye, J.; Bane, J. Role of Chest Physiotherapy in Resolving Post Operative Massive Atelectasis. *Indian Journal of Physiotherapy & Occupational Therapy* **2015**, *9*, 40-42, doi:10.5958/0973-5674.2015.00050.7.	Wrong study design
Eltorai, A.E.M.; Szabo, A.L.; Antoci, V.; Ventetuolo, C.E.; Elias, J.A.; Daniels, A.H.; Hess, D.R. Clinical Effectiveness of Incentive Spirometry for the Prevention of Postoperative Pulmonary Complications. *Respiratory Care* **2018**, *63*, 347-352, doi:10.4187/respcare.05679.	Wrong study design
Fahim, M.; Dijksman, L.M.; Derksen, W.J.M.; Bloemen, J.G.; Biesma, D.H.; Smits, A.B. Prospective Multicentre Study of a New Bowel Obstruction Treatment in Colorectal Surgery: Reduced Morbidity and Mortality. *Ejso* **2021**, *47*, 2414-2420, doi:10.1016/j.ejso.2021.05.010.	Wrong study design
Fahim, M.; Dijksman, L.M.; van Kessel, C.S.; Smeeing, D.P.J.; Braaksma, A.; Derksen, W.J.M.; Smits, A.B. Promising Results of a New Treatment in Patients with Bowel Obstruction in Colorectal Surgery. *Ejso* **2020**, *46*, 415-419, doi:10.1016/j.ejso.2019.10.011.	Wrong study design
Fiore, J.F. Use of Breathing Exercises and Enforced Mobilization after Colorectal Surgery. *Surgery* **2012**, *151*, 632-633, doi:10.1016/j.surg.2011.07.034.	Wrong study design
Ganapathy, A.; Ballard, D.H.; Bishop, G.L.; Hoegger, M.J.; Abraham, N.; D'Agostino, H.B. Pilot Study on the Influence of Incentive Spirometry on Percutaneous Image-Guided Intra-Abdominal Drainage Catheter Pressure: A Potential Method to Enhance Drainage. *Applied Sciences-Basel* **2023**, *13*, doi:10.3390/app13127308.	Wrong study design
Guerreiro Dias Rocatto, G.E.; de Carvalho Oliveira, H.J.; Semenoff Segundo, A.; Candido da Silva, A.M.; Soares Nasrala, M.L.; Nasrala Neto, E. Respiratory Physical Therapy in the Immediate Postoperative of Conventional Cholecystectomy. *Fisioterapia Brasil* **2014**, *15*, 198-202.	Wrong study design
Guinan, E.M.; Dowds, J.; Donohoe, C.; Reynolds, J.V.; Hussey, J. The Physiotherapist and the Esophageal Cancer Patient: From Prehabilitation to Rehabilitation. *Diseases of the Esophagus* **2017**, *30*, doi:10.1111/dote.12514.	Wrong study design
Güldner, A.; Pelosi, P.; de Abreu, M.G. Nonventilatory Strategies to Prevent Postoperative Pulmonary Complications. *Current Opinion in Anesthesiology* **2013**, *26*, 141-151, doi:10.1097/ACO.0b013e32835e8bac.	Wrong study design
Haapamäki, M.M.; Pihlgren, V.; Lundberg, O.; Sandzén, B.; Rutegård, J. Physical Performance and Quality of Life after Extended Abdominoperineal Excision of Rectum and Reconstruction of the Pelvic Floor with Gluteus Maximus Flap. *Diseases of the Colon & Rectum* **2011**, *54*, 101-106, doi:10.1007/DCR.0b013e3181fce26e.	Wrong study design
Honke, J.; Hiramatsu, Y.; Kawata, S.; Booka, E.; Matsumoto, T.; Morita, Y.; Kikuchi, H.; Kamiya, K.; Mori, K.; Takeuchi, H. Usefulness of Wearable Fitness Tracking	Wrong study design

Devices in Patients Undergoing Esophagectomy. *Esophagus* **2022**, *19*, 260-268, doi:10.1007/s10388-021-00893-3.	
Howard, R.; Yin, Y.S.; McCandless, L.; Wang, S.; Englesbe, M.; Machado-Aranda, D. Taking Control of Your Surgery: Impact of a Prehabilitation Program on Major Abdominal Surgery. *J Am Coll Surg* **2019**, *228*, 72-80, doi:10.1016/j.jamcollsurg.2018.09.018.	Wrong study design
Johnson, J.E.; Rice, V.H.; Fuller, S.S.; Endress, M.P. Sensory information, instruction in a coping strategy, and recovery from surgery. *Res Nurs Health* **1978**, *1*, 4-17, doi:10.1002/nur.4770010104.	Wrong study design
King, M.S. Preoperative Evaluation. *American Family Physician* **2000**, *62*, 387-396.	Wrong study design
Kuzmenko, T.S.; Vorotyntsev, S.L. Incentive Spirometry as a Way to Prevent Pulmonary Atelectasis Development. *Zaporozhye Medical Journal* **2019**, 199-202, doi:10.14739/2310-1210.2019.2.161486.	Wrong study design
Law, W.L.; Chu, K.W.; Tung, H.M. Early Outcomes of 100 Patients with Laparoscopic Resection for Rectal Neoplasm. *Surgical Endoscopy and Other Interventional Techniques* **2004**, *18*, 1592-1596, doi:10.1007/s00464-003-9249-8.	Wrong study design
Lim, L.; Lee, J.; Hwang, S.Y.; Lee, H.; Oh, S.Y.; Kang, C.; Ryu, H.G. Early Postoperative Fever and Atelectasis in Patients Undergoing Upper Abdominal Surgery. *Journal of the American College of Surgeons* **2023**, *237*, 606-613, doi:10.1097/xcs.0000000000000789.	Wrong study design
Lunardi, A.C.; Cecconello, I.; Carvalho, C.R. Postoperative chest physical therapy prevents respiratory complications in patients undergoing esophagectomy. *Rev Bras Fisioter* **2011**, *15*, 160-165, doi:10.1590/s1413-35552011000200012.	Wrong study design
Mendes, L.P.S.; Teixeira, L.S.; da Cruz, L.J.; Vieira, D.S.R.; Parreira, V.F. Sustained Maximal Inspiration Has Similar Effects Compared to Incentive Spirometers. *Respiratory Physiology & Neurobiology* **2019**, *261*, 67-74, doi:10.1016/j.resp.2019.01.006.	Wrong study design
Miskovic, A.; Lumb, A.B. Postoperative Pulmonary Complications. *British Journal of Anaesthesia* **2017**, *118*, 317-334, doi:10.1093/bja/aex002.	Wrong study design
Moorthy, K.; Halliday, L.J.; Noor, N.; Peters, C.J.; Wynter-Blyth, V.; Urch, C.E. Feasibility of Implementation and the Impact of a Digital Prehabilitation Service in Patients Undergoing Treatment for Oesophago-Gastric Cancer. *Curr Oncol* **2023**, *30*, 1673-1682, doi:10.3390/curroncol30020128.	Wrong study design
Muthu Lakshmi, T.; Rekha, K.; Saravan kumar, J.; Kabilan, R.; Preethi, G.; Yogeshwaran, L. The Effects of Early Mobilization Protocol on Pre and Post Operative Functional Performance for Individuals with Post Abdominal Surgery. *Indian Journal of Physiotherapy & Occupational Therapy* **2024**, *18*, 183-188, doi:10.37506/shbbpp68.	Wrong study design
Nevo, Y.; Shaltiel, T.; Constantini, N.; Rosin, D.; Gutman, M.; Zmora, O.; Nevler, A. Activity Tracking after Surgery: Does It Correlate with Postoperative Complications? *American Surgeon* **2022**, *88*, 226-232, doi:10.1177/0003134820988818.	Wrong study design
Olsen, M.F.; Josefson, K.; Dalenback, J.; Lundell, L.; Lonroth, H. Respiratory Function after Laparoscopic and Open Fundoplication. *European Journal of Surgery* **1997**, *163*, 667-672.	Wrong study design
Orfanos, P.; Ellis, E.; Johnston, C. Effects of Deep Breathing Exercises and Ambulation on Pattern of Ventilation in Post-Operative Patients. *Australian Journal of Physiotherapy* **1999**, *45*, 173-182, doi:10.1016/s0004-9514(14)60348-2.	Wrong study design
Overbeek, M.C.; Reijneveld, E.A.E.; Valkenet, K.; van Adrichem, E.J.; Dronkers, J.J.; Ruurda, J.P.; Veenhof, C. The Association between Preoperative Inspiratory Muscle Training Variables and Postoperative Pulmonary Complications in Subjects with Esophageal Cancer. *Respiratory Care* **2024**, *69*, 290-297, doi:10.4187/respcare.11199.	Wrong study design
Patman, S. Preoperative Physiotherapy Education Prevented Postoperative Pulmonary Complications Following Open Upper Abdominal Surgery. *Bmj Evidence-Based Medicine* **2019**, *24*, 74-75, doi:10.1136/bmjebm-2018-110985.	Wrong study design

Pfirrmann, D.; Simon, P.; Mehdorn, M.; Hänsig, M.; Stehr, S.; Selig, L.; Weimann, A.; Knödler, M.; Lordick, F.; Mehnert, A.; et al. Preconditioning Prior to Visceral Oncological Surgery. A Paradigm Shift in Visceral Surgery? *Chirurg* **2018**, *89*, 896-902, doi:10.1007/s00104-018-0709-z.	Wrong study design
Physiotherapy and Surgery. *Bandolier* **1998**, *5*, 6-6.	Wrong study design
Physiotherapy Education before Major Abdominal Surgery Reduces Lung Complications. *Frontline (20454910)* **2019**, *25*, 40-41.	Wrong study design
Platell, C.; Hall, J.C. Atelectasis after Abdominal Surgery. *Journal of the American College of Surgeons* **1997**, *185*, 584-592, doi:10.1016/s1072-7515(97)00106-3.	Wrong study design
Pontoppidan, H. Mechanical Aids to Lung Expansion in Non-Intubated Surgical Patients. *Am Rev Respir Dis* **1980**, *122*, 109-119, doi:10.1164/arrd.1980.122.5P2.109.	Wrong study design
Rakel, B.; Frantz, R. Effectiveness of Transcutaneous Electrical Nerve Stimulation on Postoperative Pain with Movement. *J Pain* **2003**, *4*, 455-464, doi:10.1067/s1526-5900(03)00780-6.	Wrong study design
Rezaiguia, S.; Jayr, C. Prevention of Respiratory Complications after Abdominal Surgery. *Annales Francaises D Anesthesie Et De Reanimation* **1996**, *15*, 623-646, doi:10.1016/0750-7658(96)82128-9.	Wrong study design
Richardson, J.; Sabanathan, S. Prevention of Respiratory Complications after Abdominal Surgery. *Thorax* **1997**, *52*, S35-S40, doi:10.1136/thx.52.2008.S35.	Wrong study design
Ruscic, K.J.; Grabitz, S.D.; Rudolph, M.I.; Eikermann, M. Prevention of Respiratory Complications of the Surgical Patient: Actionable Plan for Continued Process Improvement. *Current Opinion in Anesthesiology* **2017**, *30*, 399-408, doi:10.1097/aco.0000000000000465.	Wrong study design
Sánchez, F.G.; García, N.M. Evaluation of Postoperative Results after a Presurgical Optimisation Programme. *Perioperative Medicine* **2024**, *13*, doi:10.1186/s13741-024-00430-7.	Wrong study design
Shea, R.A.; Brooks, J.A.; Dayhoff, N.E.; Keck, J. Pain Intensity and Postoperative Pulmonary Complications among the Elderly after Abdominal Surgery. *Heart & Lung* **2002**, *31*, 440-449, doi:10.1067/mhl.2002.129449.	Wrong study design
Shimoyama, H.; Sugiyama, M.; Suzuki, Y.; Teruya, K.; Ohki, A.; Kishiki, T.; Takeuchi, H.; Sakamoto, Y.; Sunami, E.; Abe, N. Bilateral Flank Compression Maneuver for Reducing Pain on Coughing after Abdominal Surgery: A Prospective Study. *J Am Coll Surg* **2021**, *233*, 459-466.e456, doi:10.1016/j.jamcollsurg.2021.06.012.	Wrong study design
Sivabalan, T. Efficacy of Compact Disc in Abdominal Surgery. *Nursing Journal of India* **2009**, *100*, 1p-1p.	Wrong study design
Sridharan, B.; J, S.k.; Rekha, K.; Preethi, G.; Kabilan, R. Effects of Early Mobilisation on Post Operative Pulmonary Complication in Patients with Open Abdominal Surgery. *Indian Journal of Physiotherapy & Occupational Therapy* **2024**, *18*, 195-200, doi:10.37506/k5qp3907.	Wrong study design
Stiller, K. The Relationship between Post-Operative Pain and FEV Following Upper Abdominal Surgery -- a Pilot Study. *Physiotherapy Practice* **1987**, *3*, 64-69, doi:10.3109/09593988709087742.	Wrong study design
Stiller, K.R.; Munday, R.M. Chest Physiotherapy for the Surgical Patient. *British Journal of Surgery* **1992**, *79*, 745-749, doi:10.1002/bjs.1800790807.	Wrong study design
Tanaka, K.; Taoda, A.; Kashiwagi, H. Resistance Exercise after Laparoscopic Surgery Enhances Improvement in Exercise Tolerance in Geriatric Patients with Gastrointestinal Cancer. *Cureus Journal of Medical Science* **2021**, *13*, doi:10.7759/cureus.15454.	Wrong study design
Turan, A.; Khanna, A.K.; Brooker, J.; Saha, A.K.; Clark, C.J.; Samant, A.; Ozcimen, E.; Pu, X.; Ruetzler, K.; Sessler, D.I. Association between Mobilization and Composite Postoperative Complications Following Major Elective Surgery. *Jama Surgery* **2023**, *158*, 825-830, doi:10.1001/jamasurg.2023.1122.	Wrong study design
Tzani, P.; Chetta, A.; Olivieri, D. Patient Assessment and Prevention of Pulmonary Side-Effects in Surgery. *Current Opinion in Anesthesiology* **2011**, *24*, 2-7, doi:10.1097/ACO.0b013e328341abb3.	Wrong study design

van der Hulst, H.C.; Bastiaannet, E.; Portielje, J.E.A.; van der Bol, J.M.; Dekker, J.W.T. Can Physical Prehabilitation Prevent Complications after Colorectal Cancer Surgery in Frail Older Patients? *Ejso* **2021**, *47*, 2830-2840, doi:10.1016/j.ejso.2021.05.044.	Wrong study design
van der Leeden, M.; Huijsmans, R.; Geleijn, E.; de Lange-de Klerk, E.S.; Dekker, J.; Bonjer, H.J.; van der Peet, D.L. Early Enforced Mobilisation Following Surgery for Gastrointestinal Cancer: Feasibility and Outcomes. *Physiotherapy* **2016**, *102*, 103-110, doi:10.1016/j.physio.2015.03.3722.	Wrong study design
Vandenbroucque, G.; Benhamou, D.; Troche, G.; Antonello, M. Pulmonary Complication and Peritonitis: The Role of Respiratory Physiotherapy. *Medecine Et Maladies Infectieuses* **1995**, *25*, 114-120.	Wrong study design
Zeng, W.G.; Liu, M.J.; Zhou, Z.X.; Wang, Z.J. Enhanced Recovery Programme Following Laparoscopic Colorectal Resection for Elderly Patients. *ANZ J Surg* **2018**, *88*, 582-586, doi:10.1111/ans.14074.	Wrong study design

Study	Exclusion reason
Ahmed, A.; Ahmed, M.I.; Iqbal, Y.; Kamran, A.; Ahmed, M.; Ali, A. The Effect of Abdominal Binders on Mobilization and Pain in the Postoperative Period after Emergency Laparotomy. *Pakistan journal of medical and health sciences* **2022**, *16*, 785-787, doi:10.53350/pjmhs221610785.	Wrong patient population
Ali, J.; Serrette, C.; Wood, L.D.; Anthonisen, N.R. Effect of Postoperative Intermittent Positive Pressure Breathing on Lung Function. *Chest* **1984**, *85*, 192-196, doi:10.1378/chest.85.2.192.	Wrong patient population
Artıklar, T.; Erden, S. The Effect of Breathing and Coughing Exercises and Oxygen Therapy on Shoulder Pain and Analgesic Consumption after Laparoscopic Cholecystectomy: A Randomized Controlled Study. *J Perianesth Nurs* **2024**, *39*, 540-546, doi:10.1016/j.jopan.2023.10.008	Wrong patient population
Barbalho-Moulim, M.C.; Miguel, G.P.S.; Forti, E.M.P.; Campos, F.D.; Costa, D. Effects of Preoperative Inspiratory Muscle Training in Obese Women Undergoing Open Bariatric Surgery: Respiratory Muscle Strength, Lung Volumes, and Diaphragmatic Excursion. *Clinics* **2011**, *66*, 1721-1727, doi:10.1590/s1807-59322011001000009.	Wrong patient population
Bastamizad, N.; Abbasi, P.; Salari, N.; Jalali, R. Comparing the Effect of Incentive Spirometry and Deep Breathing Exercises on the Level of Shoulder Pain and Nausea Following Laparoscopic Cholecystectomy Surgery: A Clinical Trial Study. *Gastroenterology Nursing* **2023**, *46*, 14-22, doi:10.1097/SGA.0000000000000720.	Wrong patient population
Bulut, G.; Karabulut, N. The Effects of Breathing Exercises on Patients Having Laparoscopic Cholecystectomy Surgery. *Clin Nurs Res* **2023**, *32*, 805-814, doi:10.1177/10547738231154130	Wrong patient population
Casali, C.C.; Pereira, A.P.; Martinez, J.A.; de Souza, H.C.; Gastaldi, A.C. Effects of Inspiratory Muscle Training on Muscular and Pulmonary Function after Bariatric Surgery in Obese Patients. *Obes Surg* **2011**, *21*, 1389-1394, doi:10.1007/s11695-010-0349-y.	Wrong patient population
Cattano, D.; Altamirano, A.; Vannucci, A.; Melnikov, V.; Cone, C.; Hagberg, C.A. Preoperative Use of Incentive Spirometry Does Not Affect Postoperative Lung Function in Bariatric Surgery. *Translational Research* **2010**, *156*, 265-272, doi:10.1016/j.trsl.2010.08.004.	Wrong patient population
Della Via, F.; Concon Filho, A.; Kosour, C.; Ferraresi Andrade, C.E.; Guedes, E.; Dragosavac, D. Impact of Two Methods of Pulmonary Re-Expansion in Patients Undergoing Bariatric Surgery by Videolaparoscopy. *Fisioterapia Brasil* **2021**, *22*, 37-48, doi:10.33233/fb.v22i1.4387.	Wrong patient population
Eskici İlgin, V.; Yayla, A. Effect of the 4-7-8 Breathing Technique on Pain Level and Sleep Quality of Patients after Laparoscopic Bariatric Surgery: A Randomized Controlled Study. *Bariatric Surgical Practice & Patient Care* **2023**, *18*, 225-232, doi:10.1089/bari.2022.0044.	Wrong patient population

Fagevik Olsén, M.; Josefson, K.; Lönroth, H. Chest Physiotherapy Does Not Improve the Outcome in Laparoscopic Fundoplication and Vertical-Banded Gastroplasty. *Surg Endosc* **1999**, *13*, 260-263, doi:10.1007/s004649900958.	Wrong patient population
Farzanmehr, F.; Nasiri-Formi, E.; Kiany, F.; Akbari, H. Comparing the Effect of Incentive Spirometry and Deep Breathing Exercise on Hemodynamic Indicators and Pulmonary Complications During and after Gastric Sleeve Surgery. *Journal of mazandaran university of medical sciences* **2023**, *33*, 84-93.	Wrong patient population
Gastaldi, A.C.; Magalhaes, C.M.B.; Baraúna, M.A.; Silva, E.M.C.; Souza, H.C.D. Benefits of Postoperative Respiratory Kinesiotherapy Following Laparoscopic Cholecystectomy. *Brazilian Journal of Physical Therapy* **2008**, *12*, 100-106.	Wrong patient population
Hallböök, T.; Lindblad, B.; Lindroth, B.; Wolff, T. Prophylaxis against Pulmonary Complications in Patients Undergoing Gall-Bladder Surgery. A Comparison between Early Mobilization, Physiotherapy with and without Bronchodilatation. *Ann Chir Gynaecol* **1984**, *73*, 55-58.	Wrong patient population
Karthik, N.; Lodha, M.; Baksi, A.; Dutt, A.; Banerjee, N.; Swathi, M.; Choudhary, I.S.; Meena, S.P.; Sharma, N.; Puranik, A.K. Effects of Transcutaneous Electrical Nerve Stimulation on Recovery of Gastrointestinal Motility after Laparotomy: A Randomized Controlled Trial. *World Journal of Surgery* **2024**, *48*, 1626-1633, doi:10.1002/wjs.12233.	Wrong patient population
Koraş, K.; Karabulut, N. The Effect of Foot Massage on Postoperative Pain and Anxiety Levels in Laparoscopic Cholecystectomy Surgery: A Randomized Controlled Experimental Study. *Journal of perianesthesia nursing : official journal of the american society of perianesthesia nurses* **2019**, *34*, 551-558, doi:10.1016/j.jopan.2018.07.006.	Wrong patient population
Krajczy, M.; Bogacz, K.; Luniewski, J.; Szczegielniak, J. The Influence of Kinesio Taping on the Effects of Physiotherapy in Patients after Laparoscopic Cholecystectomy. *ScientificWorldJournal* **2012**, *2012*, 948282, doi:10.1100/2012/948282.	Wrong patient population
Kumar, S.; Gupta, S.; Upreti, L.; Parik, R.M. A Prospective Study to Evaluate the Effect of Deep Breathing Exercises and Incentive Spirometry on Pulmonary Functions in Patients before and after Laparoscopic Cholecystectomy. *International journal of pharmaceutical and clinical research* **2022**, *14*, 409-413.	Wrong patient population
Latha, S.; Kamath, N.; Joseph, G.; Rajesh, V. Multimodal Perioperative Management Programme on Postoperative Discomfort among Clients Undergoing Abdominal Surgery at a Selected Hospital in Mangaluru. *Biomedicine (india)* **2023**, *43*, 341-347, doi:10.51248/.v43i01.2187.	Wrong patient population
O'Connor, M.; Tattersall, M.P.; Carter, J.A. An Evaluation of the Incentive Spirometer to Improve Lung Function after Cholecystectomy. *Anaesthesia* **1988**, *43*, 785-787, doi:10.1111/j.1365-2044.1988.tb05759.x.	Wrong patient population
Pantel, H.; Hwang, J.; Brams, D.; Schnelldorfer, T.; Nepomnayshy, D. Effect of Incentive Spirometry on Postoperative Hypoxemia and Pulmonary Complications after Bariatric Surgery: A Randomized Clinical Trial. *Jama Surgery* **2017**, *152*, 422-428, doi:10.1001/jamasurg.2016.4981.	Wrong patient population
Prabhakaran, S.; Misra, S.; Magila, M.; Kumar, S.S.; Kasthuri, S.; Palanivelu, C.; Raj, P.P. Randomized Controlled Trial Comparing the Outcomes of Enhanced Recovery after Surgery and Standard Recovery Pathways in Laparoscopic Sleeve Gastrectomy. *Obes Surg* **2020**, *30*, 3273-3279, doi:10.1007/s11695-020-04585-2.	Wrong patient population
Pranavi, A.R.; Sureshkumar, S.; Mahalakshmy, T.; Kundra, P.; Kate, V. Adapted Eras Pathway Versus Standard Care in Patients Undergoing Emergency Surgery for Perforation Peritonitis-a Randomized Controlled Trial. *J Gastrointest Surg* **2022**, *26*, 39-49, doi:10.1007/s11605-021-05184-x.	Wrong patient population
Rocha, M.; Souza, S.; Costa, C.M.D.; Merino, D.F.B.; Montebelo, M.I.L.; Rasera-Júnior, I.; Pazzianotto-Forti, E.M. Airway Positive Pressure Vs. Exercises with Inspiratory Loading Focused on Pulmonary and Respiratory Muscular Functions in the Postoperative Period of Bariatric Surgery. *Arq Bras Cir Dig* **2018**, *31*, e1363, doi:10.1590/0102-672020180001e1363.	Wrong patient population

Schwieger, I.; Gamulin, Z.; Forster, A.; Meyer, P.; Gemperle, M.; Suter, P.M. Absence of Benefit of Incentive Spirometry in Low-Risk Patients Undergoing Elective Cholecystectomy. A Controlled Randomized Study. *Chest* **1986**, *89*, 652-656, doi:10.1378/chest.89.5.652.	Wrong patient population
Sim, D.T. Effectiveness of Transcutaneous Electrical Nerve Stimulation Following Cholecystectomy. *Physiotherapy* **1991**, *77*, 715-722.	Wrong patient population
Sleszynski, S.L.; Kelso, A.F. Comparison of Thoracic Manipulation with Incentive Spirometry in Preventing Postoperative Atelectasis. *J Am Osteopath Assoc* **1993**, *93*, 834-838, 843.	Wrong patient population
Svensson-Raskh, A.; Fagevik Olsen, M.; Schandl, A.; Stahle, A.; Nygren-Bonnier, M. The Respiratory Effect of Mobilization Immediately after Abdominal Surgery-a Randomized Controlled Trial. *European Respiratory Journal* **2019**, *54*, doi:10.1183/13993003.congress-2019.PA580.	Wrong patient population
Svensson-Raskh, A.; Schandl, A.R.; Ståhle, A.; Nygren-Bonnier, M.; Fagevik Olsén, M. Mobilization Started within 2 hours after Abdominal Surgery Improves Peripheral and Arterial Oxygenation: A Single-Center Randomized Controlled Trial. *Phys Ther* **2021**, *101*, doi:10.1093/ptj/pzab094.	Wrong patient population
Taylor, A.G.; Galper, D.I.; Taylor, P.; Rice, L.W.; Andersen, W.; Irvin, W.; Wang, X.Q.; Harrell, F.E. Effects of Adjunctive Swedish Massage and Vibration Therapy on Short-Term Postoperative Outcomes: A Randomized, Controlled Trial. *Journal of alternative and complementary medicine (New York, N.Y.)* **2003**, *9*, 77-89, doi:10.1089/107555303321222964.	Wrong patient population
Tenório, L.H.S.; Santos, A.C.; Neto, J.B.C.; Amaral, F.J.; Passos, V.M.M.; Lima, A.M.J.; Brasileiro-Santos, M.D. The Influence of Inspiratory Muscle Training on Diaphragmatic Mobility, Pulmonary Function and Maximum Respiratory Pressures in Morbidly Obese Individuals: A Pilot Study. *Disability and Rehabilitation* **2013**, *35*, 1915-1920, doi:10.3109/09638288.2013.769635.	Wrong patient population
Toğaç, H.K.; Yılmaz, E. Effects of Preoperative Individualized Audiovisual Education on Anxiety and Comfort in Patients Undergoing Laparoscopic Cholecystectomy: Randomised Controlled Study. *Patient Educ Couns* **2021**, *104*, 603-610, doi:10.1016/j.pec.2020.08.026.	Wrong patient population
Tomich, G.M.; Franca, D.C.; Diniz, M.T.C.; Britto, R.R.; Sampaio, R.F.; Parreira, V.F. Effects of Breathing Exercises on Breathing Pattern and Thoracoabdominal Motion after Gastroplasty. *Jornal Brasileiro De Pneumologia* **2010**, *36*, 197-204, doi:10.1590/s1806-37132010000200007.	Wrong patient population
Torrington, K.G.; Sorenson, D.E.; Sherwood, L.M. Postoperative Chest Percussion with Postural Drainage in Obese Patients Following Gastric Stapling. *Chest* **1984**, *86*, 891-895, doi:10.1378/chest.86.6.891.	Wrong patient population
Zhang, X.Y.; Wang, Q.; Zhang, S.; Tan, W.; Wang, Z.; Li, J. The Use of a Modified, Oscillating Positive Expiratory Pressure Device Reduced Fever and Length of Hospital Stay in Patients after Thoracic and Upper Abdominal Surgery: A Randomised Trial. *J Physiother* **2015**, *61*, 16-20, doi:10.1016/j.jphys.2014.11.013.	Wrong patient population
Zoremba, M.; Dette, F.; Gerlach, L.; Wolf, U.; Wulf, H. Short-Term Respiratory Physical Therapy Treatment in the Pacu and Influence on Postoperative Lung Function in Obese Adults. *Obesity Surgery* **2009**, *19*, 1346-1354, doi:10.1007/s11695-009-9922-7.	Wrong patient population

Study	Exclusion reason
Abdelaal, G.A.; Eldahdouh, S.S.; Abdelsamie, M.; Labeeb, A. Effect of Preoperative Physical and Respiratory Therapy on Postoperative Pulmonary Functions and Complications after Laparoscopic Upper Abdominal Surgery in Obese Patients. *Egyptian journal of chest diseases and tuberculosis* **2017**, *66*, 735-738, doi:10.1016/j.ejcdt.2017.10.012.	> 50% of population having surgery due to gastrointestinal cancer

Carneiro, E.M.; Ramos Mde, C.; Terra, G.A.; Rodrigues Júnior, V.; Matos, D.; Crema, E. Evaluation of Breathing Exercise in Hormonal and Immunological Responses in Patients Undergoing Abdominal Surgery. *Acta Cir Bras* **2013**, *28*, 385-390, doi:10.1590/s0102-86502013000500011.	> 50% of population having surgery due to gastrointestinal cancer
Celli, B.R.; Rodriguez, K.S.; Snider, G.L. A Controlled Trial of Intermittent Positive Pressure Breathing, Incentive Spirometry, and Deep Breathing Exercises in Preventing Pulmonary Complications after Abdominal Surgery. *Am Rev Respir Dis* **1984**, *130*, 12-15, doi:10.1164/arrd.1984.130.1.12.	> 50% of population having surgery due to gastrointestinal cancer
Christensen, E.F.; Schultz, P.; Jensen, O.V.; Egebo, K.; Engberg, M.; Grøn, I.; Juhl, B. Postoperative Pulmonary Complications and Lung Function in High-Risk Patients: A Comparison of Three Physiotherapy Regimens after Upper Abdominal Surgery in General Anesthesia. *Acta Anaesthesiol Scand* **1991**, *35*, 97-104, doi:10.1111/j.1399-6576.1991.tb03255.x.	> 50% of population having surgery due to gastrointestinal cancer
Chumillas, S.; Ponce, J.L.; Delgado, F.; Viciano, V.; Mateu, M. Prevention of Postoperative Pulmonary Complications through Respiratory Rehabilitation: A Controlled Clinical Study. *Arch Phys Med Rehabil* **1998**, *79*, 5-9, doi:10.1016/s0003-9993(98)90198-8.	> 50% of population having surgery due to gastrointestinal cancer
Condie, E.; Hack, K.; Ross, A. An Investigation of the Value of Routine Provision of Post-Operative Chest Physiotherapy in Non-Smoking Patients Undergoing Elective Abdominal Surgery. *Physiotherapy* **1993**, *79*, 547-552.	> 50% of population having surgery due to gastrointestinal cancer
Cooperman, A.M.; Hall, B.; Mikalacki, K.; Hardy, R.; Sardar, E. Use of Transcutaneous Electrical Stimulation in the Control of Postoperative Pain. *American Journal of Surgery* **1977**, *133*, 185-187, doi:10.1016/0002-9610(77)90077-0.	> 50% of population having surgery due to gastrointestinal cancer
Craven, J.L.; Evans, G.A.; Davenport, P.J.; Williams, R.H.P. The Evaluation of the Incentive Spirometer in the Management of Postoperative Pulmonary Complications. *British Journal of Surgery* **1974**, *61*, 793-797	> 50% of population having surgery due to gastrointestinal cancer
Dehghani, A.; Hajibagheri, A.; Azizi-Fini, I.; Atoof, F.; Mousavi, N. Effect of an Early Mobilisation Programme on Pain Intensity after Laparoscopic Surgery: A Randomised Clinical Trial. *Bmj Supportive & Palliative Care* **2023**, *13*, E150-E155, doi:10.1136/bmjspcare-2020-002618.	> 50% of population having surgery due to gastrointestinal cancer
Delaney, C.P.; Zutshi, M.; Senagore, A.J.; Remzi, F.H.; Hammel, J.; Fazio, V.W. Prospective, Randomized, Controlled Trial between a Pathway of Controlled Rehabilitation with Early Ambulation and Diet and Traditional Postoperative Care after Laparotomy and Intestinal Resection. *Dis Colon Rectum* **2003**, *46*, 851-859, doi:10.1007/s10350-004-6672-4.	> 50% of population having surgery due to gastrointestinal cancer
Dias Matos, F.; Santos Caracas, D.R. Impact of Positive Expiratory Pressure in Postoperative Patients After Abdominal Surgeries: A Randomized Clinical Trial. *Fisioterapia Brasil* **2019**, *20*, 610-618, doi:10.33233/fb.v20i5.2389.	> 50% of population having surgery due to gastrointestinal cancer
Forgiarini, L.A., Jr.; Carvalho, A.T.; Ferreira Tde, S.; Monteiro, M.B.; Dal Bosco, A.; Gonçalves, M.P.; Dias, A.S. Physical Therapy in the Immediate Postoperative Period after Abdominal Surgery. *J Bras Pneumol* **2009**, *35*, 455-459.	> 50% of population having surgery due to gastrointestinal cancer
Hall, J.C.; Tarala, R.A.; Tapper, J.; Hall, J.L. Prevention of Respiratory Complications after Abdominal Surgery: A Randomised Clinical Trial. *Bmj* **1996**, *312*, 148-152; discussion 152, doi:10.1136/bmj.312.7024.148.	> 50% of population having surgery due to gastrointestinal cancer
Huang, Y.T.; Lin, Y.J.; Hung, C.H.; Cheng, H.C.; Yang, H.L.; Kuo, Y.L.; Chu, P.M.; Tsai, Y.F.; Tsai, K.L. The Fully Engaged Inspiratory Muscle Training Reduces Postoperative Pulmonary Complications Rate and Increased Respiratory Muscle Function in Patients with Upper Abdominal Surgery: A Randomized Controlled Trial. *Ann Med* **2022**, *54*, 2222-2232, doi:10.1080/07853890.2022.2106511.	> 50% of population having surgery due to gastrointestinal cancer
Jung, R.; Wight, J.; Nusser, R.; Rosoff, L. Comparison of Three Methods of Respiratory Care Following Upper Abdominal Surgery. *Chest* **1980**, *78*, 31-35, doi:10.1378/chest.78.1.31.	> 50% of population having surgery due to gastrointestinal cancer
Kate, V.; Shivakumar, M.; Sureshkumar, S.; Anandhi, A.; Balakrishnan, G.; Ar, K.; Chinnakali, P.; Kundra, P. Comparison of Enhanced Recovery after Surgery (Eras) Pathway Vs. Standard Care in Patients Undergoing Elective Stoma Reversal Surgery-	> 50% of population having surgery due to gastrointestinal cancer

a Randomized Controlled Trial. *Gastroenterology* **2023**, *164*, S-1505, doi:10.1016/S0016-5085(23)04550-X.	
Labuschagne, R.; Roos, R. Pre-Operative Physiotherapy for Elderly Patients Undergoing Abdominal Surgery. *S Afr J Physiother* **2022**, *78*, 1782, doi:10.4102/sajp.v78i1.1782.	> 50% of population having surgery due to gastrointestinal cancer
Larson, C.M.; Ratzer, E.R.; Davis-Merritt, D.; Clark, J.R. The Effect of Abdominal Binders on Postoperative Pulmonary Function. *Am Surg* **2009**, *75*, 169-171.	> 50% of population having surgery due to gastrointestinal cancer
Lin, J.G.; Lo, M.W.; Wen, Y.R.; Hsieh, C.L.; Tsai, S.K.; Sun, W.Z. The Effect of High and Low Frequency Electroacupuncture in Pain after Lower Abdominal Surgery. *Pain* **2002**, *99*, 509-514, doi:10.1016/S0304-3959(02)00261-0.	> 50% of population having surgery due to gastrointestinal cancer
Lohiya, M.L.; Malviya, A.; Sharma, D.D.; Ram, S.; Chauhan, M.K.; Chauhan, L.S. Prophylactic Chest Physiotherapy in Major Abdominal Surgery among Elderly Patients. *Journal of the Indian Academy of Geriatrics* **2018**, *14*, 64-68.	> 50% of population having surgery due to gastrointestinal cancer
Madden, C.; Singer, G.; Peck, C.; Nayman, J. The Effect of Emg Biofeedback on Postoperative Pain Following Abdominal Surgery. *Anaesthesia and Intensive Care* **1978**, *6*, 333-336, doi:10.1177/0310057X7800600407.	> 50% of population having surgery due to gastrointestinal cancer
Manzano, R.M.; Carvalho, C.R.; Saraiva-Romanholo, B.M.; Vieira, J.E. Chest Physiotherapy During Immediate Postoperative Period among Patients Undergoing Upper Abdominal Surgery: Randomized Clinical Trial. *Sao Paulo Med J* **2008**, *126*, 269-273, doi:10.1590/s1516-31802008000500005.	> 50% of population having surgery due to gastrointestinal cancer
Mogan, J.; Wells, N.; Robertson, E. Effects of Preoperative Teaching on Postoperative Pain: A Replication and Expansion. *Int J Nurs Stud* **1985**, *22*, 267-280, doi:10.1016/0020-7489(85)90009-4.	> 50% of population having surgery due to gastrointestinal cancer
Mohamady, H.M.; Waked, I.S.; Attalla, A.F. Preoperative Respiratory Physical Therapy Program as a Prehabilitation to Improve Inspiratory Muscle Function and Quality of Life in Patients Undergoing Upper Abdominal Surgeries: A Prospective Randomized Controlled Trial. *Bulletin of Faculty of Physical Therapy* **2016**, *21*, 17-22.	> 50% of population having surgery due to gastrointestinal cancer
Morran, C.G.; Finlay, I.G.; Mathieson, M.; McKay, A.J.; Wilson, N.; McArdle, C.S. Randomized Controlled Trial of Physiotherapy for Postoperative Pulmonary Complications. *Br J Anaesth* **1983**, *55*, 1113-1117, doi:10.1093/bja/55.11.1113.	> 50% of population having surgery due to gastrointestinal cancer
Nichols, P.J.; Howell, B. Routine Pre- and Post-Operative Physiotherapy. Results of a Trial. *Rheumatol Phys Med* **1970**, *10*, 321-336, doi:10.1093/rheumatology/10.7.321.	> 50% of population having surgery due to gastrointestinal cancer
Ravindhran, B.; Rajan, S.; Balachandran, G.; Mohan, L.N. Do Ice Packs Reduce Postoperative Midline Incision Pain, Nsaid or Narcotic Use? *World J Surg* **2019**, *43*, 2651-2657, doi:10.1007/s00268-019-05129-1.	> 50% of population having surgery due to gastrointestinal cancer
Roukema, J.A.; Carol, E.J.; Prins, J.G. The Prevention of Pulmonary Complications after Upper Abdominal Surgery in Patients with Noncompromised Pulmonary Status. *Arch Surg* **1988**, *123*, 30-34, doi:10.1001/archsurg.1988.01400250032004.	> 50% of population having surgery due to gastrointestinal cancer
Samnani, S.S.; Umer, M.F.; Mehdi, S.H.; Farid, F.N. Impact of Preoperative Counselling on Early Postoperative Mobilization and Its Role in Smooth Recovery. *Int Sch Res Notices* **2014**, *2014*, 250536, doi:10.1155/2014/250536	> 50% of population having surgery due to gastrointestinal cancer
Simsek Yaban, Z.; Bulbuloglu, S.; Kapikiran, G.; Gunes, H.; Kula Sahin, S.; Saritas, S. The Effect of Bed Exercises Following Major Abdominal Surgery on Early Ambulation, Mobilization, Pain and Anxiety: A Randomized-Controlled Trial. *International Wound Journal* **2024**, *21*, 1-10, doi:10.1111/iwj.14406.	> 50% of population having surgery due to gastrointestinal cancer
Stein, M.; Cassara, E.L. Preoperative Pulmonary Evaluation and Therapy for Surgery Patients. *Jama* **1970**, *211*, 787-790.	> 50% of population having surgery due to gastrointestinal cancer
Stock, M.C.; Downs, J.B.; Gauer, P.K.; Alster, J.M.; Imrey, P.B. Prevention of Postoperative Pulmonary Complications with Cpap, Incentive Spirometry, and Conservative Therapy. *Chest* **1985**, *87*, 151-157, doi:10.1378/chest.87.2.151.	> 50% of population having surgery due to gastrointestinal cancer

Tyson, A.F.; Kendig, C.E.; Mabedi, C.; Cairns, B.A.; Charles, A.G. The Effect of Incentive Spirometry on Postoperative Pulmonary Function Following Laparotomy: A Randomized Clinical Trial. *JAMA Surg* **2015**, *150*, 229-236, doi:10.1001/jamasurg.2014.1846.	> 50% of population having surgery due to gastrointestinal cancer

Study	Exclusion reason
Abdikarim, I.; Cao, X.Y.; Li, S.Z.; Zhao, Y.Q.; Taupyk, Y.; Wang, Q. Enhanced Recovery after Surgery with Laparoscopic Radical Gastrectomy for Stomach Carcinomas. *World J Gastroenterol* **2015**, *21*, 13339-13344, doi:10.3748/wjg.v21.i47.13339.	Wrong intervention
Anderson, A.D.; McNaught, C.E.; MacFie, J.; Tring, I.; Barker, P.; Mitchell, C.J. Randomized Clinical Trial of Multimodal Optimization and Standard Perioperative Surgical Care. *Br J Surg* **2003**, *90*, 1497-1504, doi:10.1002/bjs.4371.	Wrong intervention
Ausania, F.; Melendez, R.; Senra, P.; Palmeiro, R.; Oubina, R.; Otero, I. Prehabilitation in Patients Undergoing Pancreaticoduodenectomy: A Randomized Controlled Trial. *Hpb* **2018**, *20*, S627-S628, doi:10.1016/j.hpb.2018.06.2205.	Wrong intervention
Boden, I.; Sullivan, K.; Hackett, C.; Winzer, B.; Hwang, R.; Story, D.; Denehy, L. Intensive Physical Therapy after Emergency Laparotomy: Pilot Phase of the Incidence of Complications Following Emergency Abdominal Surgery Get Exercising Randomized Controlled Trial. *J Trauma Acute Care Surg* **2022**, *92*, 1020-1030, doi:10.1097/ta.0000000000003542.	Wrong intervention
Boukili, I.E.; Flaris, A.N.; Mercier, F.; Cotte, E.; Kepenekian, V.; Vaudoyer, D.; Glehen, O.; Passot, G. Prehabilitation before Major Abdominal Surgery: Evaluation of the Impact of a Perioperative Clinical Pathway, a Pilot Study. *Scandinavian Journal of Surgery* **2022**, *111*, doi:10.1177/14574969221083394.	Wrong intervention
Carli, F.; Charlebois, P.; Stein, B.; Feldman, L.; Zavorsky, G.; Kim, D.J.; Scott, S.; Mayo, N.E. Randomized Clinical Trial of Prehabilitation in Colorectal Surgery. *British Journal of Surgery* **2010**, *97*, 1187-1197, doi:10.1002/bjs.7102.	Wrong intervention
Chen, B.P.; Awasthi, R.; Sweet, S.N.; Minnella, E.M.; Bergdahl, A.; Mina, D.S.; Carli, F.; Scheede-Bergdahl, C. Four-Week Prehabilitation Program Is Sufficient to Modify Exercise Behaviors and Improve Preoperative Functional Walking Capacity in Patients with Colorectal Cancer. *Supportive Care in Cancer* **2017**, *25*, 33-40, doi:10.1007/s00520-016-3379-8.	Wrong intervention
Collins, C.D.; Darke, C.S.; Knowelden, J. Chest Complications after Upper Abdominal Surgery: Their Anticipation and Prevention. *Br Med J* **1968**, *1*, 401-406, doi:10.1136/bmj.1.5589.401.	Wrong intervention
Crippa, J.; Calini, G.; Santambrogio, G.; Sassun, R.; Siracusa, C.; Maggioni, D.; Mari, G. Eras Protocol Applied to Oncological Colorectal Mini-Invasive Surgery Reduces the Surgical Stress Response and Improves Long-Term Cancer-Specific Survival. *Surg Laparosc Endosc Percutan Tech* **2023**, *33*, 297-301, doi:10.1097/sle.0000000000001181.	Wrong intervention
Dronkers, J.J.; Lamberts, H.; Reutelingsperger, I.M.; Naber, R.H.; Dronkers-Landman, C.M.; Veldman, A.; van Meeteren, N.L. Preoperative Therapeutic Programme for Elderly Patients Scheduled for Elective Abdominal Oncological Surgery: A Randomized Controlled Pilot Study. *Clin Rehabil* **2010**, *24*, 614-622, doi:10.1177/0269215509358941.	Wrong intervention
Fulop, A.; Lakatos, L.; Susztak, N.; Szijarto, A.; Banky, B. The Effect of Trimodal Prehabilitation on the Physical and Psychological Health of Patients Undergoing Colorectal Surgery: A Randomised Clinical Trial. *Anaesthesia* **2021**, *76*, 82-90, doi:10.1111/anae.15215.	Wrong intervention
García-Botello, S.; Cánovas de Lucas, R.; Tornero, C.; Escamilla, B.; Espí-Macías, A.; Esclapez-Valero, P.; Flor-Lorente, B.; García-Granero, E. Implementation of a Perioperative Multimodal Rehabilitation Protocol in Elective Colorectal Surgery. A Prospective Randomised Controlled Study. *Cir Esp* **2011**, *89*, 159-166, doi:10.1016/j.ciresp.2010.12.004.	Wrong intervention

Gawley, T.H.; Dundee, J.W. Attempts to Reduce Respiratory Complications Following Upper Abdominal Operations. *Br J Anaesth* **1981**, *53*, 1073-1078, doi:10.1093/bja/53.10.1073.	Wrong intervention
Gloor, S.; Misirlic, M.; Frei-Lanter, C.; Herzog, P.; Muller, P.; Schafli-Thurnherr, J.; Schregel, D.; Lamdark, T.; Wyss, R.; Unger, I.; et al. Prehabilitation in Patients Undergoing Colorectal Surgery Fails to Confer Reduction in Overall Morbidity: Results of a Singlecenter, Single-Blinded, Randomized Controlled Trial. *British Journal of Surgery* **2021**, *108*, iv5-iv6, doi:10.1093/bjs/znab202.015.	Wrong intervention
Hamad, A.; Zhang, H.; Huang, H.; Ejaz, A.; Tsung, A. Understanding the Mechanism Behind Preoperative Exercise Therapy in Patients with Gastrointestinal Cancers: A Prospective, Randomized Clinical Trial. *Journal of clinical oncology* **2022**, *40*, doi:10.1200/JCO.2022.40.17_suppl.LBA4087.	Wrong intervention
Ihedioha, U.; Vaughan, S.; Lloyd, G.; Mastermann, J.; Singh, B.; Chaudhri, S. Randomised Trail of Video Versus Traditional Printed Patient Education for Elective Colorectal Surgery. *Colorectal Disease* **2012**, *14*, 20, doi:10.1111/j.1463-1318.2012.03072.x.	Wrong intervention
Ihedioha, U.; Vaughan, S.; Mastermann, J.; Singh, B.; Chaudhri, S. Patient Education Videos for Elective Colorectal Surgery: Results of a Randomized Controlled Trial. *Colorectal Dis* **2013**, *15*, 1436-1441, doi:10.1111/codi.12348.	Wrong intervention
Jones, C.; Kelliher, L.; Dickinson, M.; Riga, A.; Worthington, T.; Scott, M.J.; Vandrevala, T.; Fry, C.H.; Karanjia, N.; Quiney, N. Randomized Clinical Trial on Enhanced Recovery Versus Standard Care Following Open Liver Resection. *Br J Surg* **2013**, *100*, 1015-1024, doi:10.1002/bjs.9165.	Wrong intervention
Kang, S.H.; Lee, Y.; Min, S.H.; Park, Y.S.; Ahn, S.H.; Park, D.J.; Kim, H.H. Multimodal Enhanced Recovery after Surgery (Eras) Program Is the Optimal Perioperative Care in Patients Undergoing Totally Laparoscopic Distal Gastrectomy for Gastric Cancer: A Prospective, Randomized, Clinical Trial. *Ann Surg Oncol* **2018**, *25*, 3231-3238, doi:10.1245/s10434-018-6625-0.	Wrong intervention
Kapikiran, G.; Bulbuloglu, S.; Gunes, H.; Saritas, S.; Demir, B. A Randomized Controlled Trial of Reflexology Effect after Abdominal Surgery on Patient Satisfaction, Pain and Vital Signs. *Explore* **2023**, *19*, 565-570, doi:10.1016/j.explore.2022.10.017.	Wrong intervention
Kate, V.; Kundra, P.; Swaminathan, N. Enhanced Recovery after Surgery with Respiratory Prehabilitation Versus Conventional Perioperative Protocol in Patients Undergoing Elective Gastrectomy– a Randomized Controlled Trial. *Gastroenterology* **2020**, *158*, S-1557-S-1558, doi:10.1016/S0016-5085(20)34576-5.	Wrong intervention
Khoo, C.K.; Vickery, C.J.; Forsyth, N.; Vinall, N.S.; Eyre-Brook, I.A. A Prospective Randomized Controlled Trial of Multimodal Perioperative Management Protocol in Patients Undergoing Elective Colorectal Resection for Cancer. *Ann Surg* **2007**, *245*, 867-872, doi:10.1097/01.sla.0000259219.08209.36.	Wrong intervention
Kumar, S.; Bugalia, R.P.; Meena, J. Prospective Study of Role of Multimodal Perioperative Management Protocol in Colorectal Cancer Surgery. *Journal of cardiovascular disease research* **2024**, *15*, 867-876, doi:10.48047/jcdr.2024.15.03.100.	Wrong intervention
Lee, S.M.; Kang, S.B.; Jang, J.H.; Park, J.S.; Hong, S.; Lee, T.G.; Ahn, S. Early Rehabilitation Versus Conventional Care after Laparoscopic Rectal Surgery: A Prospective, Randomized, Controlled Trial. *Surg Endosc* **2013**, *27*, 3902-3909, doi:10.1007/s00464-013-3006-4.	Wrong intervention
López-Rodríguez-Arias, F.; Sánchez-Guillén, L.; Aranaz-Ostáriz, V.; Triguero-Cánovas, D.; Lario-Pérez, S.; Barber-Valles, X.; Lacueva, F.J.; Ramirez, J.M.; Arroyo, A. Effect of Home-Based Prehabilitation in an Enhanced Recovery after Surgery Program for Patients Undergoing Colorectal Cancer Surgery During the Covid-19 Pandemic. *Support Care Cancer* **2021**, *29*, 7785-7791, doi:10.1007/s00520-021-06343-1.	Wrong intervention
Mihaljevic, A.L.Postoperative Complications and Mobilization Following Major Abdominal Surgery with Versus without Fitness Tracker-Based Feedback (Expelliarmus): A Student-Led Multicenter Randomized Controlled Clinical Trial of	Wrong intervention

the CHIR-Net SIGMA Study Group. *Annals of Surgery* **2024**, *280*, 202-211, doi:10.1097/sla.0000000000006232.	
Northgraves, M.J.; Arunachalam, L.; Madden, L.A.; Marshall, P.; Hartley, J.E.; MacFie, J.; Vince, R.V. Feasibility of a Novel Exercise Prehabilitation Programme in Patients Scheduled for Elective Colorectal Surgery: A Feasibility Randomised Controlled Trial. *Support Care Cancer* **2020**, *28*, 3197-3206, doi:10.1007/s00520-019-05098-0.	Wrong intervention
Ntoumenopoulos, G.; Greenwood, K. Effects of Cardiothoracic Physiotherapy on Intrapulmonary Shunt in Abdominal Surgical Patients. *Australian Journal of Physiotherapy* **1996**, *42*, 297-303.	Wrong intervention
Peng, L.H.; Wang, W.J.; Chen, J.; Jin, J.Y.; Min, S.; Qin, P.P. Implementation of the Pre-Operative Rehabilitation Recovery Protocol and Its Effect on the Quality of Recovery after Colorectal Surgeries. *Chin Med J (Engl)* **2021**, *134*, 2865-2873, doi:10.1097/cm9.0000000000001709.	Wrong intervention
Qi, S.; Chen, G.D.; Cao, P.; Hu, J.P.; He, G.S.; Luo, J.X.; He, J.; Peng, X.D. Safety and Efficacy of Enhanced Recovery after Surgery (Eras) Programs in Patients Undergoing Hepatectomy: A Prospective Randomized Controlled Trial. *Journal of Clinical Laboratory Analysis* **2018**, *32*, doi:10.1002/jcla.22434.	Wrong intervention
Sabajo, C.R.; ten Cate, D.W.G.; Heijmans, M.H.M.; Koot, C.T.G.; van Leeuwen, L.V.L.; Slooter, G.D. Prehabilitation in Colorectal Cancer Surgery Improves Outcome and Reduces Hospital Costs. *Ejso* **2024**, *50*, doi:10.1016/j.ejso.2023.107302	Wrong intervention
Schuppisser, J.P.; Brändli, O.; Meili, U. Postoperative Intermittent Positive Pressure Breathing Versus Physiotherapy. *Am J Surg* **1980**, *140*, 682-686, doi:10.1016/0002-9610(80)90058-6.	Wrong intervention
Shen, Y.; Chen, X.; Hou, J.; Chen, Y.; Fang, Y.; Xue, Z.; D'Journo, X.B.; Cerfolio, R.J.; Fernando, H.C.; Fiorelli, A.; et al. The Effect of Enhanced Recovery after Minimally Invasive Esophagectomy: A Randomized Controlled Trial. *Surg Endosc* **2022**, *36*, 9113-9122, doi:10.1007/s00464-022-09385-6.	Wrong intervention
Soares, S.M.; Nucci, L.B.; da Silva, M.M.; Campacci, T.C. Pulmonary Function and Physical Performance Outcomes with Preoperative Physical Therapy in Upper Abdominal Surgery: A Randomized Controlled Trial. *Clin Rehabil* **2013**, *27*, 616-627, doi:10.1177/0269215512471063	Wrong intervention
Swaminathan, N.; Kundra, P.; Ravi, R.; Kate, V. Eras Protocol with Respiratory Prehabilitation Versus Conventional Perioperative Protocol in Elective Gastrectomy- a Randomized Controlled Trial. *Int J Surg* **2020**, *81*, 149-157, doi:10.1016/j.ijsu.2020.07.027.	Wrong intervention
Taha, A.; Taha-Mehlitz, S.; Staartjes, V.E.; Lunger, F.; Gloor, S.; Unger, I.; Mungo, G.; Tschuor, C.; Breitenstein, S.; Gingert, C. Association of a Prehabilitation Program with Anxiety and Depression before Colorectal Surgery: A Post Hoc Analysis of the Peracs Randomized Controlled Trial. *Langenbecks Archives of Surgery* **2021**, *406*, 1553-1561, doi:10.1007/s00423-021-02158-0.	Wrong intervention
Ten Cate, D.W.G.; Molenaar, C.J.L.; Garcia, R.S.; Bojesen, R.D.; Tahasildar, B.L.R.; Jansen, L.; López-Baamonde, M.; Feo, C.V.; Martínez-Palli, G.; Gögenur, I.; et al. Multimodal Prehabilitation in Elective Oncological Colorectal Surgery Enhances Postoperative Functional Recovery: A Secondary Analysis of the Prehab Randomized Clinical Trial. *Eur J Surg Oncol* **2024**, *50*, 108270, doi:10.1016/j.ejso.2024.108270.	Wrong intervention
Tian, Y.; Cao, S.; Liu, X.; Li, L.; He, Q.; Jiang, L.; Wang, X.; Chu, X.; Wang, H.; Xia, L.; et al. Randomized Controlled Trial Comparing the Short-Term Outcomes of Enhanced Recovery after Surgery and Conventional Care in Laparoscopic Distal Gastrectomy (Gissg1901). *Ann Surg* **2022**, *275*, e15-e21, doi:10.1097/sla.0000000000004908.	Wrong intervention
Triguero-Cánovas, D.; López-Rodríguez-Arias, F.; Gómez-Martínez, M.; Sánchez-Guillén, L.; Peris-Castelló, F.; Alcaide-Quirós, M.J.; Morillas-Blasco, P.; Arroyo, A.; Ramírez, J.M. Home-Based Prehabilitation Improves Physical Conditions Measured by Ergospirometry and 6mwt in Colorectal Cancer Patients: A Randomized	Wrong intervention

Controlled Pilot Study. *Support Care Cancer* **2023**, *31*, 673, doi:10.1007/s00520-023-08140-4.	
Wang, Q.; Suo, J.; Jiang, J.; Wang, C.; Zhao, Y.Q.; Cao, X. Effectiveness of Fast-Track Rehabilitation Vs Conventional Care in Laparoscopic Colorectal Resection for Elderly Patients: A Randomized Trial. *Colorectal Dis* **2012**, *14*, 1009-1013, doi:10.1111/j.1463-1318.2011.02855.x.	Wrong intervention
Wang, W.K.; Tu, C.Y.; Shao, C.X.; Chen, W.; Zhou, Q.Y.; Zhu, J.D.; Xu, H.T. Impact of Enhanced Recovery after Surgery on Postoperative Rehabilitation, Inflammation, and Immunity in Gastric Carcinoma Patients: A Randomized Clinical Trial. *Braz J Med Biol Res* **2019**, *52*, e8265, doi:10.1590/1414-431x20198265.	Wrong intervention
Welsch, T.; Wolk, S.; Linke, S.; Mussle, B.; Bogner, A.; Meissner, T.; Wierick, A.; Weitz, J.; Rahbari, N.N.; Distler, M.; et al. Use of Activity Tracking in Major Visceral Surgery-the Enhanced Perioperative Mobilization (Epm) Trial: A Randomized Controlled Trial. *European surgical research. Europaische chirurgische Forschung. Recherches chirurgicales europeennes* **2018**, *59*, 6, doi:10.1159/000488177.	Wrong intervention
Yamana, I.; Takeno, S.; Hashimoto, T.; Maki, K.; Shibata, R.; Shiwaku, H.; Shimaoka, H.; Shiota, E.; Yamashita, Y. Randomized Controlled Study to Evaluate the Efficacy of a Preoperative Respiratory Rehabilitation Program to Prevent Postoperative Pulmonary Complications after Esophagectomy. *Dig Surg* **2015**, *32*, 331-337, doi:10.1159/000434758.	Wrong intervention
Zelic, M.; Sotosek-Tokmadzic, V.; Uravic, M.; Sustic, A. Multimodal Rehabilitation with Pre and Early Postoperative Feeding and Early Mobilization Improves Recovery after Laparoscopic Colorectal Surgery. *Surgical Endoscopy and Other Interventional Techniques* **2014**, *28*, S74, doi:10.1007/s00464-014-3484-z.	Wrong intervention

Study	Exclusion reason
Balvardi, S.; Pecorelli, N.; Castelino, T.; Niculiseanu, P.; Alhashemi, M.; Liberman, A.S.; Charlebois, P.; Stein, B.; Carli, F.; Mayo, N.E.; et al. Impact of Facilitation of Early Mobilization on Postoperative Pulmonary Outcomes after Colorectal Surgery: A Randomized Controlled Trial. *Ann Surg* **2021**, *273*, 868-875, doi:10.1097/sla.0000000000003919.	Wrong comparator
Begum, S.; Waseem, M.; Ahmed, F.; Agarwal, A.; Ahmad, S.; Ahmad, F. A Comparative Study between Thera Pep and Incentive Spirometer in Upper Abdominal Surgery Patients. *Indian Journal of Physiotherapy & Occupational Therapy* **2010**, *4*, 147-149.	Wrong comparator
Campbell, T.; Ferguson, N.; McKinlay, R.G.C. The Use of a Simple Self-Administered Method of Positive Expiratory Pressure (Pep) in Chest Physiotherapy after Abdominal Surgery. *Physiotherapy* **1986**, *72*, 498-500.	Wrong comparator
Cheifetz, O.; Lucy, S.D.; Overend, T.J.; Crowe, J. The Effect of Abdominal Support on Functional Outcomes in Patients Following Major Abdominal Surgery: A Randomized Controlled Trial. *Physiotherapy Canada* **2010**, *62*, 242-253, doi:10.3138/physio.62.3.242.	Wrong comparator
Chen, J.; Peng, L.H.; Min, S. Implementation of Perioperative Breathing Exercises and Its Effect on Postoperative Pulmonary Complications and Long-Term Prognosis in Elderly Patients Undergoing Laparoscopic Colorectal Surgery: A Randomized Controlled Trial. *Clin Rehabil* **2022**, *36*, 1229-1243, doi:10.1177/02692155221097762.	Wrong comparator
Czyżewski, P.; Szczepkowski, M.; Domaniecki, J.; Dąbek, A. Physiotherapy Based on Pnf Concept for Elderly People after Conventional Colon Surgery. *Pol Przegl Chir* **2013**, *85*, 475-482, doi:10.2478/pjs-2013-0074.	Wrong comparator
de Almeida, E.P.M.; de Almeida, J.P.; Landoni, G.; Galas, F.; Fukushima, J.T.; Fominskiy, E.; de Brito, C.M.M.; Cavichio, L.B.L.; de Almeida, L.A.A.; Ribeiro, U., Jr.; et al. Early Mobilization Programme Improves Functional Capacity after Major Abdominal Cancer Surgery: A Randomized Controlled Trial. *Br J Anaesth* **2017**, *119*, 900-907, doi:10.1093/bja/aex250.	Wrong comparator

Dias, C.M.; Plácido, T.R.; Ferreira, M.F.B.; Guimaraes, F.S.; Menezes, S.L.S. Incentive Spirometry and Breath Stacking:: Effects on the Inspiratory Capacity of Individuals Submitted to Abdominal Surgery. *Brazilian Journal of Physical Therapy* **2008**, *12*, 94-99.	Wrong comparator
Elay, G.; Kaya, S.; Yildirim, A.; Coskun, R.; Gundogan, K.; Akbudak, I.H.; Guven, M.; Sungur, M. Has High-Frequency Oscillation Technique Any Benefit to Prevent Pulmonary Complications in Abdominal Surgery Patients? *Journal of medical and surgical intensive care medicine* **2015**, *6*, 65-69, doi:10.5152/dcbybd.2015.804.	Wrong comparator
Fagevik Olsén, M.; Becovic, S.; Dean, E. Short-Term Effects of Mobilization on Oxygenation in Patients after Open Surgery for Pancreatic Cancer: A Randomized Controlled Trial. *BMC Surg* **2021**, *21*, 185, doi:10.1186/s12893-021-01187-2.	Wrong comparator
Fernandes, D.D.L.; Righi, N.C.; Rubin Neto, L.J.; Bellé, J.M.; Pippi, C.M.; Ribas, C.; Nichele, L.F.I.; Signori, L.U.; Silva, A. Effects of the Breath Stacking Technique after Upper Abdominal Surgery: A Randomized Clinical Trial. *J Bras Pneumol* **2022**, *48*, e20210280, doi:10.36416/1806-3756/e20210280	Wrong comparator
Fernandes, S.C.; Carnieli-Cazati, D.; Timenetsky, K.T.; Giovanetti, E.A.; Caserta, R.A. Intermittent Positive Pressure and Volume Respiratory Incentivator Improves Lung Function in Abdominal Surgical Patients. *Intensive Care Medicine* **2014**, *40*, S27, doi:10.1007/s00134-013-3451-5.	Wrong comparator
Fiore, J.F.; Castelino, T.; Pecorelli, N.; Niculiseanu, P.; Balvardi, S.; Hershorn, O.; Liberman, S.; Charlebois, P.; Stein, B.; Carli, F.; et al. Ensuring Early Mobilization within an Enhanced Recovery Program for Colorectal Surgery <I>a Randomized Controlled Trial</I>. *Annals of Surgery* **2017**, *266*, 223-231, doi:10.1097/sla.0000000000002114.	Wrong comparator
Giannini, A.; Palini, G.M.; Ferri, N.; Veneroni, L. Enhanced Abdominal Wall Rehabilitation after Major Surgery: A Randomized Controlled Trial. *Chirurgia* **2016**, *29*, 114-120.	Wrong comparator
Guinan, E.M.; Forde, C.; O'Neill, L.; Gannon, J.; Doyle, S.L.; Valkenet, K.; Trappenburg, J.C.A.; van Hillegersberg, R.; Ravi, N.; Hussey, J.M.; et al. Effect of Preoperative Inspiratory Muscle Training on Physical Functioning Following Esophagectomy. *Dis Esophagus* **2019**, *32*, doi:10.1093/dote/doy091.	Wrong comparator
Hall, J.C.; Tarala, R.; Harris, J.; Tapper, J.; Christiansen, K. Incentive Spirometry Versus Routine Chest Physiotherapy for Prevention of Pulmonary Complications after Abdominal Surgery. *Lancet* **1991**, *337*, 953-956, doi:10.1016/0140-6736(91)91580-n.	Wrong comparator
Heisterberg, L.; Johansen, T.S.; Larsen, H.W.; Holm, M.; Andersen, B. Postoperative Pulmonary Complications in Upper Abdominal Surgery. A Randomized Clinical Comparison between Physiotherapy and Blow-Bottles. *Acta Chir Scand* **1979**, *145*, 505-507	Wrong comparator
Kapre, V.M.; Shukla, M. A Comparative Study of Flow and Volume Oriented Incentive Spirometry after Upper Abdominal Surgery Using Pft Studies. *Indian Journal of Physiotherapy & Occupational Therapy* **2015**, *9*, 93-98, doi:10.5958/0973-5674.2015.00020.9.	Wrong comparator
Kizmaz, E.; Gursoy, S.; Atalay, O.T.; Sungurtekin, U. Expiratory Muscle Training Versus Incentive Spirometry after Colorectal Surgery Pulmonary Rehabilitation after Colorectal Surgery. *Annals of Clinical and Analytical Medicine* **2023**, *14*, 286-290, doi:10.4328/ACAM.21451.	Wrong comparator
Kumar, A.S.; Alaparthi, G.K.; Augustine, A.J.; Pazhyaottayil, Z.C.; Ramakrishna, A.; Krishnakumar, S.K. Comparison of Flow and Volume Incentive Spirometry on Pulmonary Function and Exercise Tolerance in Open Abdominal Surgery: A Randomized Clinical Trial. *J Clin Diagn Res* **2016**, *10*, Kc01-06, doi:10.7860/jcdr/2016/16164.7064.	Wrong comparator
Kumar, S. Comparison of Flow and Volume Incentive Spirometry on Pulmonary Function and Exercise Tolerance in Open Abdominal Surgery-a Randomized Clinical Trial. *European Respiratory Journal* **2016**, *48*, doi:10.1183/13993003.congress-2016.PA4429.	Wrong comparator

Lunardi, A.; Paisani, D.; da Silva, C.M.; Cano, D.; Carvalho, C. Effect of Lung Expansion Techniques in the Incidence of Pulmonary Complications after Upper Abdominal Surgery. *European Respiratory Journal* **2013**, *42*.	Wrong comparator
Maity, B.; Choudhury, A.; Banerjee, C. The Efficacy of Incentive Spirometry in Restoring Baseline Lung Capacity after Upper Abdominal and Midline Laparotomy: A Randomised Case Control Study. *International journal of academic medicine and pharmacy* **2023**, *5*, 1961-1966, doi:10.47009/jamp.2023.5.3.386.	Wrong comparator
Min, J.; An, K.Y.; Park, H.; Cho, W.; Jung, H.J.; Chu, S.H.; Cho, M.; Yang, S.Y.; Jeon, J.Y.; Kim, N.K. Postoperative Inpatient Exercise Facilitates Recovery after Laparoscopic Surgery in Colorectal Cancer Patients: A Randomized Controlled Trial. *BMC Gastroenterol* **2023**, *23*, 127, doi:10.1186/s12876-023-02755-x.	Wrong comparator
Minschaert, M.; Vincent, J.L.; Ros, A.M.; Kahn, R.J. Influence of Incentive Spirometry on Pulmonary Volumes after Laparotomy. *Acta Anaesthesiol Belg* **1982**, *33*, 203-209.	Wrong comparator
Naz, S.; Siddiqui, F.A.; Ikram, S. Effectiveness of the Incentive Spirometry in Preventing Post-Operative Pulmonary Complications after Laparotomy. *Rawal medical journal* **2014**, *39*, 274-276.	Wrong comparator
Nirali, M.; Srivastava, S. Added Effect of Deep Breathing and Diaphragmatic Breathing Exercise in Upper Abdominal Surgery Patients: A Randomised Clinical Trial. *Indian journal of public health research and development* **2020**, *11*, 544-549, doi:10.37506/v11/i2/2020/ijphrd/194861.	Wrong comparator
Olsen, M.F.; Josefson, K.; Wiklund, M. Evaluation of Abdominal Binder after Major Upper Gastrointestinal Surgery. *Advances in Physiotherapy* **2009**, *11*, 104-110, doi:10.1080/14038190802141073.	Wrong comparator
Pereira, M.G.; Silva, A.M.O.; Galhardo, F.D.M.; Almeida, B.D.M.; Lopes, R.L.; Boin, I. Respiratory Muscle Training with Electronic Devices in the Postoperative Period of Hepatectomy: A Randomized Study. *World J Hepatol* **2023**, *15*, 688-698, doi:10.4254/wjh.v15.i5.688.	Wrong comparator
Ricksten, S.E.; Bengtsson, A.; Soderberg, C.; Thorden, M.; Kvist, H. Effects of Periodic Positive Airway Pressure by Mask on Postoperative Pulmonary Function. *Chest* **1986**, *89*, 774-781, doi:10.1378/chest.89.6.774.	Wrong comparator
Rowley, D.D.; Malinowski, T.P.; Di Peppe, J.L.; Sharkey, R.M.; Gochenour, D.U.; Enfield, K.B. A Randomized Controlled Trial Comparing Two Lung Expansion Therapies after Upper Abdominal Surgery. *Respir Care* **2019**, *64*, 1181-1192, doi:10.4187/respcare.06812.	Wrong comparator
Schrempf, M.C.; Zanker, J.; Arndt, T.T.; Vlasenko, D.; Anthuber, M.; Müller, G.; Sommer, F.; Wolf, S. Immersive Virtual Reality Fitness Games to Improve Recovery after Colorectal Surgery: A Randomized Single Blind Controlled Pilot Trial. *Games Health J* **2023**, *12*, 450-458, doi:10.1089/g4h.2023.0004.	Wrong comparator
Silva, Y.R.; Li, S.K.; Rickard, M.J. Does the Addition of Deep Breathing Exercises to Physiotherapy-Directed Early Mobilisation Alter Patient Outcomes Following High-Risk Open Upper Abdominal Surgery? Cluster Randomised Controlled Trial. *Physiotherapy* **2013**, *99*, 187-193, doi:10.1016/j.physio.2012.09.006.	Wrong comparator
Syropoulos, S.; Kalogeropoulos, A.; Maniatis, G.; Iliodromiti, Z. The Effect of Respiratory Physiotherapy on Pain and Pulmonary Complications in Major Upper Abdominal Surgery. *Archives of hellenic medicine* **2016**, *32*, 724-731.	Wrong comparator
Taha, M.M.; Draz, R.S.; Gamal, M.M.; Ibrahim, Z.M. Adding Autogenic Drainage to Chest Physiotherapy after Upper Abdominal Surgery: Effect on Blood Gases and Pulmonary Complications Prevention. Randomized Controlled Trial. *Sao Paulo Med J* **2021**, *139*, 556-563, doi:10.1590/1516-3180.2021.0048.0904221.	Wrong comparator
Trevisan, M.E.; Soares, J.C.; Rondinel, T.Z. Effects of Two Respiratory Incentive Techniques on Chest Wall Mobility after Upper Abdominal Surgery. *Fisioterapia e Pesquisa* **2010**, *17*, 322-326.	Wrong comparator
Valkenet, K.; Trappenburg, J.C.A.; Ruurda, J.P.; Guinan, E.M.; Reynolds, J.V.; Nafteux, P.; Fontaine, M.; Rodrigo, H.E.; van der Peet, D.L.; Hania, S.W.; et al. Multicentre Randomized Clinical Trial of Inspiratory Muscle Training Versus Usual	Wrong comparator

Care before Surgery for Oesophageal Cancer. *British Journal of Surgery* **2018**, *105*, 502-511, doi:10.1002/bjs.10803.	
Wilnerzon Thörn, R.M.; Forsberg, A.; Stepniewski, J.; Hjelmqvist, H.; Magnuson, A.; Ahlstrand, R.; Ljungqvist, O. Immediate Mobilization in Post-Anesthesia Care Unit Does Not Increase Overall Postoperative Physical Activity after Elective Colorectal Surgery: A Randomized, Double-Blinded Controlled Trial within an Enhanced Recovery Protocol. *World J Surg* **2024**, *48*, 956-966, doi:10.1002/wjs.12102.	Wrong comparator
Zhao, C.H.; Sun, Y.H.; Mao, X.M. Volume Incentive Spirometry Reduces Pulmonary Complications in Patients after Open Abdominal Surgery: A Randomized Clinical Trial. *Int J Gen Med* **2023**, *16*, 793-801, doi:10.2147/ijgm.S400030.	Wrong comparator

Study	Exclusion reason
Boden, I.; El-Ansary, D.; Zalucki, N.; Robertson, I.K.; Browning, L.; Skinner, E.H.; Denehy, L. Physiotherapy Education and Training Prior to Upper Abdominal Surgery Is Memorable and Has High Treatment Fidelity: A Nested Mixed-Methods Randomised-Controlled Study. *Physiotherapy* **2018**, *104*, 194-202, doi:10.1016/j.physio.2017.08.008.	Wrong outcome
Clay, L.; Gunnarsson, U.; Franklin, K.A.; Strigård, K. Effect of an Elastic Girdle on Lung Function, Intra-Abdominal Pressure, and Pain after Midline Laparotomy: A Randomized Controlled Trial. *Int J Colorectal Dis* **2014**, *29*, 715-721, doi:10.1007/s00384-014-1834-x.	Wrong outcome
Hardy, E.J.; Hatt, J.; Doleman, B.; Smart, T.F.; Piasecki, M.; Lund, J.N.; Phillips, B.E. Post-Operative Electrical Muscle Stimulation Attenuates Loss of Muscle Mass and Function Following Major Abdominal Surgery in Older Adults: A Split Body Randomised Control Trial. *Age and Ageing* **2022**, *51*, doi:10.1093/ageing/afac234.	Wrong outcome
Tokuda, M.; Tabira, K.; Masuda, T.; Nishiwada, T.; Shomoto, K. Effect of Modulated-Frequency and Modulated-Intensity Transcutaneous Electrical Nerve Stimulation after Abdominal Surgery: A Randomized Controlled Trial. *Clin J Pain* **2014**, *30*, 565-570, doi:10.1097/AJP.0b013e31829ea151.	Wrong outcome
Vats, N. Effect of Deep Breathing Exercises and Incentive Spirometry in the Prevention of Post Operative Pulmonary Complications in the Patients of Cancer Esophagus Undergoing Esophagectomy. *Indian Journal of Physiotherapy & Occupational Therapy* **2009**, *3*, 60-67.	Wrong outcome

Study	Exclusion reason
Koo, M.; Hwang, S. [Comparison of Effects of Exhalation and Inhalation Breathing Exercises on Pulmonary Function and Complications in Elderly Patients with Upper-Abdominal Surgery]. *J Korean Acad Nurs* **2016**, *46*, 514-522, doi:10.4040/jkan.2016.46.4.514.	Study not in English (unable to translate to English)
Li, Y.; Qiu, J.; Cao, H. [Application of Enhanced Recovery after Surgery for Patients with Laparoscopic Radical Gastrectomy]. *Zhonghua Wei Chang Wai Ke Za Zhi* **2016**, *19*, 269-273.	Study not in English (unable to translate to English)
Proske, J.M.; Raue, W.; Neudecker, J.; Müller, J.M.; Schwenk, W. [Fast Track Rehabilitation in Colonic Surgery: Results of a Prospective Trial]. *Ann Chir* **2005**, *130*, 152-156, doi:10.1016/j.anchir.2004.12.012.	Study not in English (unable to translate to English)
Paphalmi, R.; Ipcsics, C.; Antony More, P. Chest Physiotherapy and Respiratory-Circulatory Training During the Preoperative and Postoperative Periods of Abdominal Surgery. *Nővér* **1995**, *8*, 16-18.	Study not in English (unable to translate to English)
Pelliccioni, M.; Bertozzi, L.; Giannini, A.; Veneroni, L.; De Luigi, F. Study of the Effectiveness of Physiotherapy Treatment on Patients Exposed to Major Abdominal Surgery. *Scienza Riabilitativa* **2010**, *12*, 7-13.	Study not in English (unable to translate to English)
Lim, C.; Cho, H.; Chang, S.H. The Effect of Deep Breathing Exercise and Incentive Spirometry to Prevent Postoperative Pulmonary Complications after Abdominal	Study not in English (unable to translate to English)

Surgery in Geriatric Patients. *Korean Journal of Anesthesiology* **1997**, *33*, 1185-1191.	
Sun, Y.C.; Sun, Z.Q.; Wang, Q.S. Application of Eras of Colorectal Cancer Patients in Perioperative Period. *Chinese journal of cancer prevention and treatment* **2018**, *25*, 494-498.	Study not in English (unable to translate to English)
