# Supplementary material for: Interventions Provided by Physiotherapists to Prevent Complications After Major Gastrointestinal Cancer Surgery: A Systematic Review and Meta-Analysis
Source: Cancers (Basel). 2025 Feb 17;17(4):676. doi: 10.3390/cancers17040676 (PMC11853706; doi:10.3390/cancers17040676)
Supplement: Supplementary file 1 [file cancers-17-00676-s001.zip › SR Supplementary material V2 SW.pdf]

Systematic Review Supplementary Material

Table S1: Detailed inclusion and exclusion criteria

Population	Included	Excluded
Patient	Adults 18 years and over	Under 18 years of age
Elective/ Emergency	Elective or emergency surgery	Excluding if gastrointestinal surgery performed in context of emergency multi-trauma - orthopaedic/ multi-trauma/ spinal/ neuro
Setting/ timing for intervention	Pre-operatively – primary care/ in hospital<br>Post-operatively – interventions performed in the acute hospital/ inpatients.	If intervention was performed post discharge from hospital
Surgery type	Major gastrointestinal surgery (colorectal, upper GI, hepatobiliary)<br><br>Intra-peritoneal operation, no primary involvement of thorax, involves luminal resection and/ or resection of solid organ associated with gastrointestinal tract<br><br>General anaesthetic<br><br>At least 24 hours/ overnight stay	Not major gastrointestinal surgery according to Courtney 2024 [12] and Boden 2024 [6] definition<br>PLUS<br>Exclude if cohort contains more than 10% of procedures classified as minor by the authors and specifically excluded – total abdominal hysterectomy, appendectomies. Only include if data for “major” surgery sub cohort can be extracted from these papers.<br><br>Exclude if surgical cohort is exclusively cholecystectomy (laparoscopic or open)<br>Transplant surgery (liver, bowel, kidney, pancreas)

	No less than 50% of patients in study must be having surgery due to gastrointestinal cancer	If unable to determine that > 50% of study participants are having surgery due to gastrointestinal cancer.
Incision type	Any incision type (open, laparoscopic, robotic, hand assisted)	Exclude gastroscopy/ colonoscopy/ endoscopy
Intervention (respiratory physiotherapy)	Included	Excluded
Professional	The intervention is designed and delivered by:<br>Physiotherapy<br>Physical therapist<br>Respiratory therapists<br>Kinesiologists<br>Kinesiotherapists<br>**OR** an Allied Health Assistant under explicit instruction from the above professions	Disciplines other than the listed.
Intent of intervention	Interventions are delivered with the intent of prophylaxis against complications	Where the intervention is delivered in response to signs of deterioration/ complications or to treat a diagnosed PPC
Physiotherapy interventions	Education specific to respiratory complication prophylaxis and early mobilisation.	Education for obstetric/ antenatal surgery<br><br>Prehabilitation that is whole body strength and/ or conditioning (any modality) that has intent of enhancing exercise tolerance

	Patient education and training to perform self-directed breathing exercises<br><br>Lung expansion exercises with or without the use of devices as augmentation<br>- Incentive spirometry (IS)<br>- Positive expiratory pressure (PEP) (any form)<br>- Respiratory muscle training<br>- Coached/ supervised breathing exercises<br><br>Interventions delivered with prophylactic intent<br><br>Mobilisation - Upright mobility (out of bed) for the intent of exercise (supervised or assisted) and physical activity<br><br>Respiratory muscle training alone (respiratory prehabilitation) without whole body strength/ conditioning<br><br>Multimodal delivery of combination of eligible	Where the eligible physiotherapy intervention is delivered as part of a multi-modal package of care and the effect of the physiotherapy intervention alone cannot be determined<br><br>High flow nasal prongs in absence of physiotherapy intervention<br><br>Where the intervention is non-invasive ventilation (NIV)<br><br>Where interventions are performed on predominantly mechanically ventilated patients (>60%)<br><br>Interventions where mobility is considered exercises in bed/ seated on the bedside or mobility is for the purposes of hygiene rather than exercise<br><br>Respiratory muscle training combined with whole body exercise training.

	interventions only eg. Preoperative education AND lung expansion exercises with device AND mobility	
Comparator		
Interventions compared to nothing or usual care	True no treatment control or usual care of a culture of mobilisation and general encouragement of breathing exercises but not coached or the use of respiratory devices	Where interventions are compared to other active interventions such as coached deep breathing, incentive spirometry
Outcomes		
Pulmonary complications	Primary outcome – post operative pulmonary complication as defined by the primary trial and could include the following<br>- composite score eg. Melbourne Group Score<br>- atelectasis (as diagnosed from CXR, CT, or lung ultrasound), pneumonia (any diagnostic construct), acute respiratory failure (any diagnostic construct), acute hypoxemia (arterial blood gases, pulse oximetry), or composite PPC diagnostic tools (any diagnostic construct)	Measures of spirometry or lung function only<br>If the only outcome reported for PPC was pneumothorax, pleural effusion or bronchospasm

Quality of life	As defined by individual papers	
Functional status	As defined by individual papers	
Discharge destination	As defined by individual papers	
Health economics	As defined by individual papers	
Length of stay	Length of stay (acute care)	
Medical complications	Medical complications as defined by Molenaar [37]	
Surgical complications	Surgical complications as defined by Molenaar [37]	
Gastrointestinal complications	As reported by individual study	
All cause complications	Claven-dindo classification and grading and as a proportion of the group	
Adverse events related to physiotherapy intervention	As reported by individual study	
Mortality (all cause) - 30 day	As reported by individual study	

Table S2: Medline via PubMed Search Strategy

#1	"Respiratory Therapy"[MeSH] OR "Spirometry"[MeSH] OR "Exercise Movement Techniques"[Mesh] OR "Breathing Exercises" [Mesh] OR "Exercise Therapy"[Mesh] OR "Physical Therapy Modalities"[Mesh:NoExp] OR "Preoperative Exercise"[Mesh] OR "Early Ambulation"[Mesh] OR "Walking" [Mesh] OR physiotherapy[Title/Abstract] OR physical therapy[Title/Abstract] OR physical therapist[Title/Abstract] OR respiratory therapy[Title/Abstract] OR respiratory therapist[Title/Abstract] OR kinesiotherapy[Title/Abstract] OR patient education[Title/Abstract] OR deep breathing exercises[Title/Abstract] OR lung expansion exercises[Title/Abstract] OR thoracic expansion exercises[Title/Abstract] OR coughing[Title/Abstract] OR incentive spirometry[Title/Abstract] OR respiratory devices[Title/Abstract] OR positive expiratory pressure[Title/Abstract] OR inspiratory muscle training[Title/Abstract] OR prehabilitation[Title/Abstract] OR prehab[Title/Abstract] OR pre-habilitation[Title/Abstract] OR mobilization[Title/Abstract] OR mobility[Title/Abstract] OR early mobility[Title/Abstract] OR mobilisation[Title/Abstract] OR exercise[Title/Abstract] OR ambulation [Title/Abstract]
#2	"Abdomen/surgery"[Mesh] OR "Digestive System Surgical Procedures"[Mesh] OR "Colorectal surgery" [Mesh] OR "Laparotomy" [Mesh] OR "Surgical Oncology" [Mesh] OR "Abdominal Neoplasms" [Mesh] OR "Digestive System Neoplasms" [Mesh] OR abdominal surgery[Title/Abstract] OR visceral surgery[Title/Abstract] OR cholecystectomy[Title/Abstract] OR gastrectomy[Title/Abstract] OR colorectal surgery[Title/Abstract] OR hepatobiliary surgery[Title/Abstract] OR liver surgery[Title/Abstract] OR pancreas surgery[Title/Abstract] OR upper gastrointestinal surgery[Title/Abstract] OR laparotomy[Title/Abstract] OR colectomy[Title/Abstract] OR bowel resection[Title/Abstract]
#3	(complication* [Title/Abstract] OR pneumonia [Title/Abstract] OR atelectasis [Title/Abstract] OR hypoxaemia [Title/Abstract] OR "Postoperative Complications"[Mesh] OR "Pneumonia"[Mesh] OR "Pulmonary Atelectasis" [Mesh])
#4	#1 AND #2 AND #3
#5	((randomized controlled trial [pt] OR controlled clinical trial [pt] OR randomized [tiab] OR placebo [tiab] OR drug therapy [sh] OR randomly [tiab] OR trial [tiab] OR groups [tiab]) NOT (animals [mh] NOT humans [mh]))
#6	#4 AND #5

Table S4: Cochrane Risk of Bias Assessment

	D1	D2	D3	D4	D5	Overall
Boden 2018	+	+	+	+	+	+
Mackay 2005	+	!	+	+	+	+
Lunardi 2015	+	!	+	!	+	!
Boden 2021	+	+	+	+	+	+
Fagevik Olsen 1997	+	+	+	!	!	!
Anh 2013	+	+	+	+	+	+
Qin 2020	+	+	+	!	+	!
Singh 2023	!	-	+	-	!	-
Kulkarni 2010	+	+	+	-	-	-

+ Low risk

! Some concerns

\- High risk

D1 Randomisation process

D2 Deviations from the intended interventions

D3 Missing outcome data

D4 Measurement of the outcome

D5 Selection of the reported result

Table S5: Classification of data extracted for all cause complications per study (excluding postoperative pulmonary complications):

**Study**	**Complications**
**Boden 2018 [25]**	Extracted from Table 2: Postoperative clinical events and complications. Included all identified complications *except* surgical lacerations and acute respiratory failure
**Mackay 2005 [28]**	Extracted from Table 5: Clinical outcomes - Postoperative complications other than pulmonary complications – identified in text as predominantly wound infection, nausea and vomiting, cardiac failure, atrial fibrillation
**Lunardi 2015 [30]**	Extrapolated from in-text reporting of deaths from cardiac complications
**Boden 2021 [26]**	N/A – already accounted for in primary analysis Boden 2018
**Fagevik Olsen 1997 [29]**	No reporting made of postoperative complications other than postoperative pulmonary complications
**Ahn 2013 [31]**	Extrapolated from in-text reporting of one postoperative wound infection and one postoperative ileus
**Kulkarni 2010 [27]**	No reporting made of postoperative complications other than postoperative pulmonary complications
**Qin 2021 [32]**	Extracted from Table 3: Excluded already accounted for PPCs of pneumonia, atelectasis and respiratory failure and planned return to surgery.

**Singh 2023 [33]**	No reporting made of postoperative complications other than postoperative pulmonary complications
